# Profound Changes in Net Energy and Nitrogen Metabolites Fluxes within the Splanchnic Area during Overfeeding of Yucatan Mini Pigs That Remain Euglycemic

**DOI:** 10.3390/nu11020434

**Published:** 2019-02-19

**Authors:** Isabelle Savary-Auzeloux, Ahmed-Ben Mohamed, Benoit Cohade, Dominique Dardevet, Jérémie David, Noureddine Hafnaoui, Carole Migné, Estelle Pujos-Guillot, Didier Rémond, Sergio Polakof

**Affiliations:** 1 Université Clermont Auvergne, INRA, UNH, Unité de Nutrition Humaine, CRNH Auvergne, F-63000 Clermont Ferrand, France; ahmed-ben.mohamed@inra.fr (A.-B.M.); benoit.cohade@inra.fr (B.C.); dominique.dardevet@clermont.inra.fr (D.D.); jeremie.david@inra.fr (J.D.); noureddine.hafnaoui@inra.fr (N.H.); didier.remond@inra.fr (D.R.); sergio.polakof@inra.fr (S.P.); 2 Université Clermont Auvergne, INRA, UNH, Unité de Nutrition Humaine, PFEM, Metabo-Hub Clermont, CRNH Auvergne, F-63000 Clermont Ferrand, France; carole.migne@inra.fr (C.M.); estelle.pujos-guillot@inra.fr (E.P.-G.)

**Keywords:** liver, gut, obesity, amino acid, glucose, lactate, nutrient flux, short chain fatty acid, minipig

## Abstract

A dysregulation of nutrient exchange between tissues (gut, liver, muscles, adipose) occurs during overnutrition and could induce obesity and metabolic diseases. We aimed to evaluate how, in overfed mini pigs, nutrients use and partition were regulated in the gut and liver. Net nutrients fluxes were assessed in the fed (PP) and post absorptive (PA) states at 1, 14 and 60 days of adaptation to overfeeding in five adult Yucatan female multicatheterized minipigs. Pigs PA glycaemia and PP-induced hyperglycemia remained unchanged over the experimental period, suggesting that the management of the excess of energy intake allowed the maintenance of glucose levels. This was associated with (1) an increased PA plasma insulin, (2) an increased gut lactate production (increased lactate net release +89%, 1 h PP, D1 vs. D60) probably from an increased glucose oxidation, (3) a shift in utilization of gluconeogenic precursor (lactate, propionate) in the liver, and (4) a reduced gut utilization of nitrogen moieties for energy purposes (glutamine), a nitrogen sparing effect at the whole body level (decreased plasma urea in PA (−24% D1 vs. D60) and PP states) and a specific increased level of AA involved in lipids handling and bile recycling in the gut lumen (taurine and glycine).

## 1. Introduction

Prevalence of overweight and obesity has increased dramatically over the last 35 years, as well as the associated chronic diseases (diabetes, cardiovascular diseases, cancers) [1]. Obesity results from an imbalance between energy supply and expenditure due to increased food intake and reduced physical activity. This imbalance, when occurring over a long period of time, induces metabolic dysregulations leading firstly to metabolic adaptations capable to maintain normal homeostatic state and later to the development of metabolic disturbances such as low grade inflammation [2], insulin resistance [3] and, ultimately, pathologies. These late perturbations occur when tissues and organs are no longer capable to deal with the nutrient oversupply and maintain homeostasis compatible with a healthy status.

The most deleterious effects of over-nutrition on tissues and organs are observed on liver (development of non-alcoholic fatty liver disease) [4], subcutaneous and visceral adipose tissues [5], muscle [6], inflammatory cells [7], etc. An altered gut profile of bacteria species [8] and richness [9] have also been demonstrated in obese or overfed or diabetic humans and animals, which explains the increasing interest on the impact of the gut (and its tightly connected microbiota) on the development of insulin resistance and associated pathologies. Consequently, the gut-liver axis has recently regained increasing interest [10]. Interestingly, it has been shown that the splanchnic area (and particularly the liver) was metabolically disturbed early (i.e., within the first days) in streptozotocin treated rats [11,12]. These disturbances at the splanchnic level occur before dysfunctions of peripheral tissues are demonstrated. However, the interaction between gut and liver, although considered to be one of the keys in the development of hepatic and whole body obesity-related pathologies [13,14,15], has been relatively scarcely studied due to a lack of access to metabolites/factors present the portal vein which is the only vascular link from the gut to the liver. In addition, the investigation of the gut-liver axis is complex due to the combination of various metabolically intertwined tissues and organs in this area (microbiota, gut, liver, visceral adipose tissues). On the top of this, the investigation of the adaptation mechanisms stimulated to deal with excess of nutrients is also complex because gut and liver, but also nutrients and metabolites are at the crossroad of various metabolic pathways leading to a competition between tissues for metabolites utilization [14,16,17].

Consequently, from this analysis of the data obtained so far in the field, we focused on the investigation of (1) the gut-liver nutrients utilization within the first steps of obesity/IR development in a situation of overfeeding and (2) the major routes of exchange of nutrients between gut, liver, and the periphery during obesity development. This approach will help to decipher what the gut actually releases (as nutrients and signals) to the liver, what is taken up/released to the liver, and what is finally available to peripheral tissues in the early phases of adaptation to overfeeding and obesity development.

To address the point concerning the early steps of obesity development, we have recently investigated the whole body and tissues metabolic adaptations by analyzing genes expressions and activities of enzymes (in the fed state) involved in the major metabolic pathways of nutrients utilization/synthesis in gut, liver, and adipose and muscle tissues in Yucatan mini pigs overfed for two months with a high fat, high sucrose diet [18,19,20]. We have confirmed specific inter-organ metabolic adaptations developed by the pigs to handle the sudden and massive dietary supply of nutrients and we have shown the development of an obese phenotype (rapid weight gain, increased cholesterol, and insulin plasma levels in the fasted state), as well as a reduced potential to phosphorylate glucose, a decreased capacity for de novo lipogenesis and an increased arterial insulin and branched chain plasma amino acids. The present work aims at investigating the adaptive dynamics of the net fluxes of energy and nitrogenous nutrients (i.e., the consequence of the genes regulation) between tissues in the splanchnic area both in the fasted and the fed states. The main objective is to understand how the gut and the liver net uptake and release of major nutrients/metabolites in a situation of overfeeding affects nutrients available to peripheral tissues.

To address this issue, multicatheterized (in artery, portal vein, and hepatic vein) Yucatan minipigs were overfed for a period of two months as previously published [18,19]. Net fluxes of energy and nitrogenous metabolites were measured across the gut and the liver in the fasted and fed states over the several weeks of overfeeding.

## 2. Materials and Methods

### 2.1. Animals and Experimental Procedure

The study involved five female adult Yucatan mini-pigs (30 ± 1 kg). Three weeks before the experimentation, the mini-pigs were surgically fitted with permanent catheters (polyvinyl chloride; 1.1 mm i.d., 1.9 mm o.d.) in the abdominal aorta, the portal vein, and the sus hepatic vein for blood sampling and in mesenteric vein for infusions. The animals were housed in subject pens (1 × 1.5 m) in a ventilated room with controlled temperature (21 °C) and regular light cycle (L12:D12). They were fed once daily with 400 g/d of a concentrate feed containing 17.5% protein, 3.2% fat, 4.3% cellulose, and 5.2% ash (Porcyprima; Sanders Centre Auvergne, Aigueperse, France) and had free access to tap water. Catheters were flushed and filled with a saline solution containing heparin (1/10) three times a week over the experimental period to avoid the formation of blood clots in the catheters. All procedures were in accordance with the guidelines formulated by the European Community for the use of experimental animals (L358-86/609/EEC, Council Directive, 1986; authorization 02090.01).

At least two weeks after surgery, the mini-pigs were fed a High Fat High Sugar (HFHS) diet consisting in a regular pig diet enriched with fat (13% palm oil) and sugar (10% sucrose) (1 kg/day, 13.3 kJ/day) for two months. Lipids represent 27% of energy supply in the diet. This diet was offered in two meals (500*g* each meal) at 8:00 am and 16:00 pm. Over the entire experimental period, the animals ingested their meal in no more than 10 min and no refusal was observed. Samplings were performed on D1 (1st day of HFHS exposure), D14 and D60. After an overnight fast, blood samples were simultaneously withdrawn from the aorta, the portal vein and the sus-hepatic vein on heparinized or EDTA treated tubes, before the meal, 30, 60, 120, 180, 240, 330, 420 and 510 min post-meal ingestion. Blood was centrifuged at 4500 × *g* for 10 min, plasma rapidly collected and stored at −80 °C until further analyses. Body weight was determined weekly.

For measurement of plasma flow, a solution of 0.185 M of sodium p-aminohippurate (PAH) (pH 7.4) was infused in the mesenteric vein at a rate of 12 mL/h. The infusion started 1 h before feeding and lasted over the entire post prandial sampling period. The plasma flows in each vessel was calculated according to the Fick principle [21].

After the two-month experimental period, the mini-pigs were euthanized after an overnight fast by intravenous administration of Dolethal^®^ (pentobarbitone sodium 200 mg/L, Vetoquinol^®^, Magny-Vernois, France).

### 2.2. Analytical Procedures

Glucose, lactate, urea, triacylglycerol (TG), High Density Lipoprotein (HDL)-cholesterol, Low Density Lipoprotein (LDL)-cholesterol, total cholesterol, albumin, aspartate aminotransferase (AST), alanine aminotransferase (ALT), and fructosamine concentrations were enzymatically measured using commercial kits on an automotive ABX Pentra 400 (Horiba Medical, Montpellier, France) test system. Plasma insulin levels were assayed using a commercial ELISA kit (Mercodia, Uppsala, Sweden). Data at D1 and D60 for glucose, urea, triacylglycerol (TG), HDL-cholesterol, LDL-cholesterol, total cholesterol, and insulin in artery have already been published [18]. Data for these parameters at D14 have been added to evaluate the short-term vs. longer term kinetics of adaptation to overfeeding.

For amino acid measurements, detailed procedures are described elsewhere [22]. In brief, plasma samples were deproteinised with sulphosalicylic acid after adding norleucine as an internal standard. The supernatant was diluted (2/3) with a lithium injection buffer containing glucosaminic acid as an injection standard and amino acids concentrations were determined with an amino acid analyzer (Hitachi L8900, Sciencetec, Villebon/Yvette, France) by ion exchange chromatography with postcolumn derivatisation with ninhydrine.

PAH concentration in plasma was measured according to [23], 250 µL of plasma samples were deproteinized with sulphosalicylic acid acid, thoroughly mixed and centrifuged at 10,000× *g*, 4 °C, for 15 min. 80 µL of the supernanant was deacetylated by adding 20 µL of 5 M HCL, followed by an incubation at 90 °C for 1 h [24]. Sodium nitrite (625 mg/L) was then added manually. The samples were then inserted into the automotive ABX Pentra 400 (Horiba Medical, Montpellier, France) which added successively ammonium sulfamate (0.64 g/L) and *N*-(1-Naphtyl) ethylenediamine dihydrochloride (1 mg/mL). Concentrations were determined by comparison with PAH standard and read out at 600 nm.

Short chain fatty acids (SCFA) in plasma were measured using 1-(tert-butyldimethylsilyl) imidazole (MTBSTFA) derivatization and analysis by gas chromatography (GC) according to [25]. Shortly, to 500 µL of plasma was added 50 µL of a mixture of 13C labelled 1-^13^C-acetate (4 mM), 1-^13^C-propionate (1.5 mM), 1-^13^C-butyrate (0.6 mM) (Cortecnec, Voisins Le Bretonneux, France), and 10 µL of 37% (*v*/*v*) HCL solution. A total of 2 mL of diethyl ether was added to plasma and the mixture was centrifuged (10 min, 2000 rpm). A total of 50 µL of MTBSTFA (Tokyo Chemical Industry, Tokyo, Japan) was added to supernatant for SCFA derivatization. The mixture was injected in GC–MS system 7890A (Agilent Technologies California, USA) using the splitless mode equipped with a quadrupole detector (5975C) and autoinjector (7683). The ionisation mode was operated in electron impact (electron energy 70 eV). The GC system was fitted with a nonpolar capillary column DB-5 MS (J&W Scientific, Folsom, CA, USA, 30 m × 0.25 mm i.d. × 0.25 μm film thickness) for chromatographic separation. Quantification of the SCFA was performed using the Selected Ion Monitoring acquisition Mode by measurement of the m/z ratios of the specific ^13^C and ^12^C ions of each quantified SCFA and comparison to a standard curve: 117/118 (^12^C acetate/1-^13^C-acétate), 131/132 (^12^C propionate/1-^13^C-propionate), 145/146 (^12^C butyrate/1-^13^C-butyrate). SCFA content in the feces was determined by NMR following a water extraction of feces samples.

### 2.3. Calculations

The HOMA2-IR (Homeostasis Model Assessment 2, Insulin-Resistance) was calculated from arterial insulin and glucose levels by the program HOMA Calculator v2.2.3 (The Oxford Centre for Diabetes, Endocrinology and Metabolism. Diabetes Trials Unit, Oxford University, Oxford, United Kingdom; http://www.dtu.ox.ac.uk/ToolsSoftware/).

Net nutrient fluxes through the gut (viscera drained by the portal vein), the liver and total splanchnic tissues (gut + liver) were calculated as described by Katz et al. [21]. The net nutrient fluxes were calculated as differences between the efferent flux and the afferent flux. Consequently, a positive net flux indicates a net release whereas a negative net flux indicates a net uptake.

Metabolite (MET) net flux across the portal-drained viscera was calculated as follows: ([MET]_PV_-[MET]_A_) × PF_PV_ where PF_PV_ is the portal plasma flow and [MET]_PV,A_ the plasma concentrations of the metabolite in the portal vein and the aorta, respectively. The net hepatic flux of metabolites was calculated as follows: ([MET]_HV_ × PF_HV_ − ([MET]_PV_ × PF_PV_ + [MET]_A_ × PF_AH_) where [MET]_HV, PV and A_ are the plasma concentrations of the metabolite in the hepatic vein, portal vein and aorta, respectively and PF _HV, PV and A_ are the plasma flows in the hepatic vein, portal vein and artery, respectively. Lastly, the net flux of AA and urea across overall splanchnic tissues was calculated as follows: ([MET]_HV_-[MET]_A_) × PF_HV_ where PF_HV_ is the plasma flow in the hepatic vein and [MET]_HV and A_, the metabolite concentrations in the hepatic vein and artery. Indispensable amino acids (IAA) included histidine, isoleucine, leucine, lysine, methionine, phenylalanine, threonine, tryptophane, and valine; non-indispensable amino acids (NIAA) included alanine, glutamate, glutamine, glycine, serine, tyrosine, cysteine, citrulline, ornithine, arginine, proline, 3-methylhistidine; and branched-chain amino acids (BCAA) included isoleucine, leucine, and valine. Total amino acids (TAA) were the sum of IAA and NIAA.

### 2.4. Statistics

All data are expressed as means ± SEM. Comparisons of data between D0, D14, and D60 in the fasted state were performed using a one way repeated measures ANOVA (SigmaPlot 12, Systat software, San Jose, CA, USA) followed by a post hoc analysis using the Holm Sidak test. Comparison of data between D1, D14, and D60 (adaptation to the diet) and between hours post test meal ingestion in the fed state (t0 (fasted), t30, t60, t120, t180, t240, t330, t420, t510 min post meal ingestion) were performed using a two way repeated measures ANOVA and followed by post hoc analysis using the Holm Sidak test. Differences were considered significant if *p* < 0.05 and as a tendency (t) for 0.05 < *p* < 0.1.

## 3. Results

### 3.1. Overfeeding, Impact on Pigs’ Weight, Insulin Levels, Alteration of Energy Nutrients Concentrations and Net Splanchnic Uptake in the Fasted and Fed States: Data Presented in Table 1, Table 2, Table 3, Table 4 and Table 5 and Figure 6 (for Insulin)

#### 3.1.1. Energy Nutrients

Overfeeding led to a 43% increase of pigs’ weight over the 60 days of the experimental period (*p* < 0.05). At the plasma level and as already observed in a similar model of overfed animals [18], lactate, total plasma cholesterol, HDL cholesterol, and LDL cholesterol were significantly (*p* < 0.05) or tended to be increased over the experimental period (+54%, +44%, +58%, D60 vs. D1 for lactate, total and HDL cholesterol, respectively; +34% D14 vs. D1 for LDL cholesterol) whereas no effect on glucose, triglycerides, albumin, alanine aminotransferase, and aspartate aminotransferase was observed in the fasted state at D14 and D60 relatively to D1 (Table 1). The stable glucose arterial levels observed between D1 and D60, suggests that animals were still capable to maintain their glycaemia although glucose content in the diet (supplied as saccharose or starch) and net plasma gut release of glucose is higher at D60 vs. D14 and D1 in the fasted state after adaptation to overfeeding (4.9× between D1 and D60, *p* = 0.06, Table 4). This active glucose utilization by the liver (net splanchnic release in the fasted state not significantly altered between D1, D14 and D60, Table 4) can be associated with the increased insulin levels (*p* < 0.05), as well as HOMA index (Figure 6) in the artery at D14 and D60.

Lactate levels were severely altered in the fasted state, as previously shown in a study in a similar model [18]: arterial, portal and hepatic vein concentrations increased significantly at D14 and D60 vs. D1 (+34% and +89% at D60 vs. D1 in the portal and hepatic vein, respectively, *p* < 0.05, data not shown). Only an increased net hepatic lactate release between D1 and D60 was observed (*p* = 0.03) (Table 4).

Fasting arterial short chain fatty acids levels (acetate + propionate + butyrate: C2 + C3 + C4) were increased (+37%, D1 vs. D60, *p* < 0.05), essentially due to an increased acetate level (+38%, D1 vs. D60, *p* < 0.05), which is the only major SCFA significantly altered at the arterial level (Table 2). Minor SCFA (isobutyrate and isovalerate) were also significantly modified over the experimental period: their concentration decreased between D1 and D14 and significantly re-increased between D14 and D60 (+32% for isobutyrate and +25% for isovalerate between D14 and D60) (Table 2). Except for fasting propionate in the hepatic vein that tend to be increased at D60 (+23% D60 vs. D1 *p* = 0.06, Table 2), the SCFA levels in the hepatic vein are not significantly modified. However, they tended to be increased in the portal vein (+18%, D60 vs. D1 for C2 + C3 + C4, *p* = 0.09, Table 2). Concerning the net overall splanchnic release of SCFA (Table 4), it was not modified by overfeeding even if the net gut release of butyrate (+43%, D60 vs. D1, *p* = 0.05) and to a lesser extend propionate (+33%, D60 vs. D1, *p* = 0.09) increased over the experimental period. Quantitatively, it also should be noted that except for acetate, the net splanchnic release represents only a small percentage of what is released by the gut: 3%, 27%, 7%, 11% at D1 for propionate, butyrate, isobutyrate and isovalerate, respectively, implying an important utilization of these molecules by the liver, whatever the diet (Table 4).

#### 3.1.2. Nitrogenous Nutrients

Urea levels were strongly decreased in the fasted state in all blood vessels at D14 (−39%, −26% and −27% at D14 vs. D0 in artery, portal vein and hepatic vein respectively, *p* < 0.05), and to a lesser extent at D60 (Table 1). The small differences in concentrations between vessels did not allow us to determine significant variations in net gut or hepatic uptake/release of urea throughout the experimental period (Table 4) although sites or urea recycling and synthesis are mainly located at the splanchnic level. Arterial BCAA, and particularly leucine levels, were significantly increased between D1 and D60 (+14% and +18% for BCAA and leucine, respectively, Table 3), as already shown in a previous paper, using other methodologies for assessment of total BCAA levels [19]. Other AA, such as tryptophan, tended to increase (+28% D60 vs. D1, *p* = 0.09) as well as non-indispensable AA, such as glycine (+26%, D60 vs. D1, *p* = 0.01), proline (+76%, D60 vs. D1, *p* = 0.003), and serine (+24%, D60 vs. D1, *p* = 0.09) (Table 3). Taken together, arterial NIAA and TAA (but not IAA) were increased at D60 vs. D1 (+26% and +17% for arterial NIAA and TAA, *p* = 0.03 and *p* = 0.07, respectively). On the contrary, methionine, and to a lesser extent phenylalanine, decreased over the same period (−28% (*p* = 0.02) and −17% (*p* = 0.06) between D1 and D14 for methionine and phenylalanine respectively). Except for methionine and phenylalanine, the significant increased arterial concentration observed for leucine, isoleucine, valine, tryptophane, glycine, serine, proline, NIAA, and TAA was (or tended to be) present also in the portal and hepatic veins (Table 3).

Although concentrations of several AA were modified between D1 and D60, an absence of impact of adaptation to overfeeding on nearly all the net gut, hepatic and splanchnic amino acids fluxes in the fasted state (Table 5) were observed. Only an increased glutamate net gut uptake at D14 (+ 180%, *p* < 0.05), a tendency for an increased glycine release by the gut at D14 and D60 (+339% at D14, *p* < 0.1) and alterations of tryptophan net plasma gut release and liver uptake (*p* < 0.05) occurred.

### 3.2. Overfeeding, Impact on Pigs’ Weight, Insulin Levels, Alteration of Energy Nutrient Concentrations, and Net Splanchnic Uptake in the Fed State. Data Presented in Figure 1, Figure 2, Figure 3, Figure 4, Figure 5 and Figure 6

#### 3.2.1. Energy Nutrients

**Figure 1:**
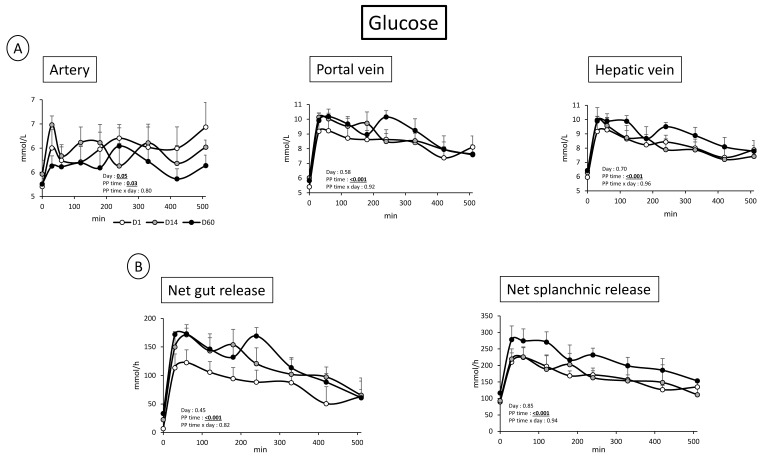
(**A**) Glucose concentration (mmol/L) after meal intake (post prandial state, PP: 0 to 510 min) in artery, portal vein, and hepatic vein. (**B**) Net glucose gut and splanchnic (gut + liver) fluxes (mmol/h) after meal intake (Post prandial state, PP: 0 to 510 min) before (D1) and after 14 days (D14) and 60 days (D60) of adaptation to HFHS diet. Two-way RM ANOVA, * D14 significantly different from D1, † D60 significantly different from D1, ‡ D60 significantly different from D14; Values are means ± SEM. For calculations and statistical treatment of the data and for details, see Materials and Methods.

Even if arterial, portal, and sus-hepatic vein glucose concentrations were increased after meal ingestion (PP time < 0.05), the duration of adaptation to overfeeding (D1 vs. D14 vs. D60) did not modify the plasma postprandial glucose profile in artery, portal, or hepatic veins (Figure 1). These data are consistent with what found in the fasted state. No significant “day effect” was found concerning postprandial glucose net gut and splanchnic release (Figure 1).

**Figure 2:**
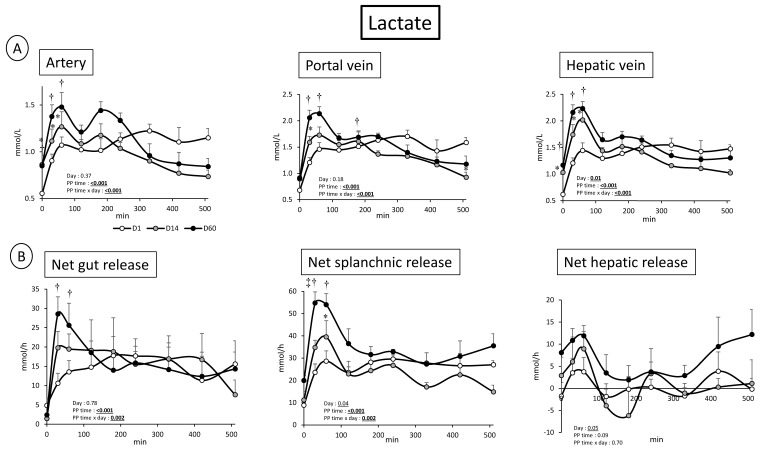
(**A**) Lactate concentration (mmol/L) after meal intake (Post prandial state, PP: 0 to 510 min) in artery, portal vein and hepatic vein; and (**B**) net lactate gut, splanchnic (gut + liver) and hepatic fluxes (mmol/h) after meal intake (post prandial state, PP: 0 to 510 min) before (D1) and after 14 days (D14) and 60 days (D60) of adaptation to HFHS diet. Two way RM ANOVA, * D14 significantly different from D1, † D60 significantly different from D1, ‡ D60 significantly different from D14; Values are means ± SEM. For calculations and statistical treatment of the data and for details, see Materials and Methods.

**Figure 3:**
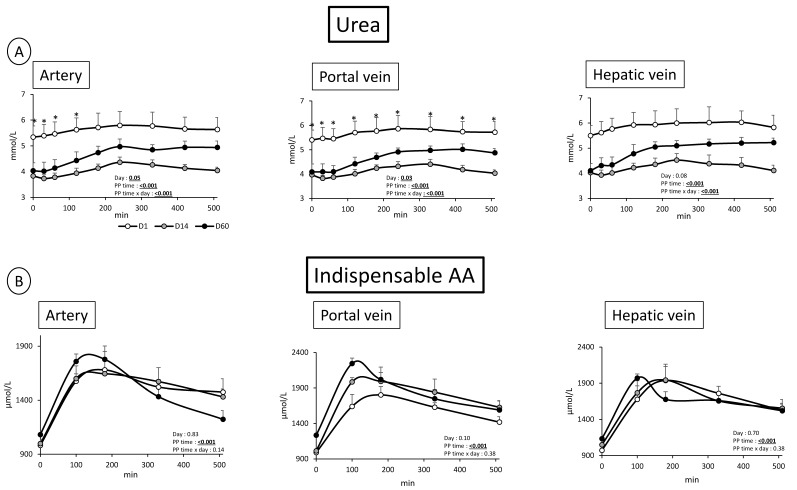
(**A**) Urea concentration (mmol/L) after meal intake (Post prandial state, PP: 0 to 510 min) in artery, portal vein and hepatic vein; (**B**) indispensable amino acids (AA) concentration (µmol/L) after meal intake (Post prandial state, PP: 0 to 510 min) in artery, portal vein and hepatic vein; before (D1) and after 14 days (D14) and 60 days (D60) of adaptation to HFHS diet. Two way RM ANOVA, * D14 significantly different from D1, † D60 significantly different from D1, ‡ D60 significantly different from D14; Values are means ± SEM. For calculations and statistical treatment of the data and for details, see Materials and Methods.

On the contrary, and consistently to what observed in the fasted state, arterial plasma as well as portal and hepatic veins lactate concentration were more elevated 60 min after feeding at D60 compared to D1 (PP time × day effect: *p* < 0.001 in the three vessels, Figure 3). This was accompanied with a significant (PP time × Day: *p* < 0.002) increased net gut and splanchnic release of lactate. Lastly, insulin was increased post-prandially (particularly 30 min after meal ingestion) to a similar extend at D1 and D60 whereas this increase was of a greater importance at D14 (day effect: 0.03) (Figure 2).

#### 3.2.2. Nitrogenous Nutrients

Similarly to what observed in the fasted state, urea levels were lower in the fed state in all blood vessels at D14 (*p* < 0.05), and to a lesser extent at D60 (Figure 3). Again, the small differences in concentrations between vessels did not allow us to determine significant variations in net gut or hepatic uptake/release of urea throughout the experimental period.

**Figure 4:**
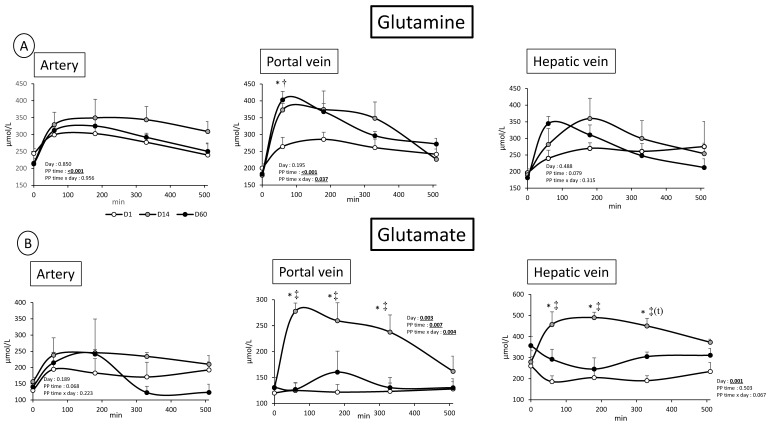
(**A**) Glutamine concentration (µmol/L) after meal intake (post prandial state, PP: 0 to 510 min) in artery, portal vein and hepatic vein; and (**B**) glutamate concentration (µmol/L) after meal intake (post prandial state, PP: 0 to 510 min) in artery, portal vein and hepatic vein; before (D1) and after 14 days (D14) and 60 days (D60) of adaptation to HFHS diet. Two way RM ANOVA, * D14 significantly different from D1, † D60 significantly different from D1, ‡ D60 significantly different from D14; Values are means ± SEM. For calculations and statistical treatment of the data and for details, see Materials and Methods.

**Figure 5:**
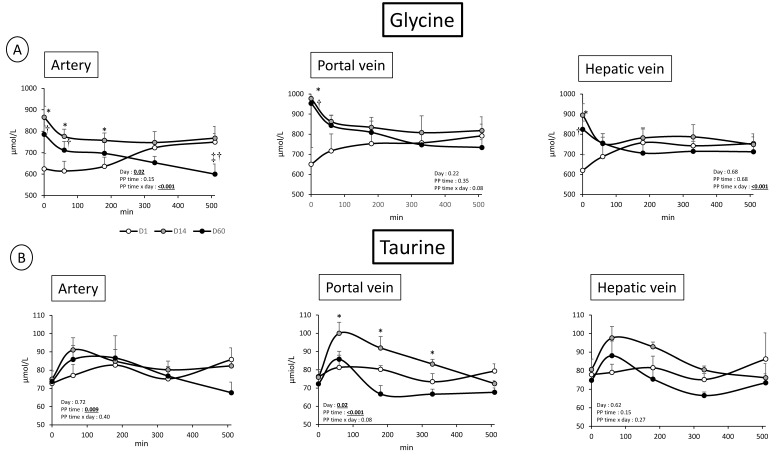
(**A**) Glycine concentration (µmol/L) after meal intake (Post prandial state, PP: 0 to 510 min) in artery, portal vein and hepatic vein; and (**B**) taurine concentration (µmol/L) after meal intake (post prandial state, PP: 0 to 510 min) in artery, portal vein and hepatic vein; before (D1) and after 14 days (D14) and 60 days (D60) of adaptation to HFHS diet. Two way RM ANOVA, * D14 significantly different from D1, † D60 significantly different from D1, ‡ D60 significantly different from D14; Values are means ± SEM. For calculations and statistical treatment of the data and for details, see Materials and Methods.

In the fed state, and as could be expected, a significant (PP time effect: *p* < 0.01) increased TAA, IAA and NIAA concentration in the 3 vessels was observed with a peak 1 and 3 h post meal ingestion (IAA data shown in Figure 3). In artery, only glycine and proline levels were not increased or did not tend to be altered by meal ingestion (Figure 5, data not shown for Proline). Similarly to many other AA, glutamine was increased in the fed state in all vessels (PP time effect: *p* < 0.001, *p* < 0.001, and *p* = 0.079 in artery, portal vein, and hepatic vein, respectively) (Figure 4).

However, an important increased glutamine level in the portal vein was observed at D14 and D60 relatively to D1 (PP time × day effect = 0.037) whereas PP effect is lower (*p* = 0.08) in hepatic vein (Figure 4), suggesting an increased release by the gut associated with an increased utilization by the liver. Glutamate followed the same pattern of change in the portal vein as glutamine, but only at D14 (day effect: *p* = 0.003, PP time × day effect: 0.004) (D1 and D60 similar) (Figure 5). However, the increased glutamate concentration observed in the portal vein was also present in the hepatic vein (day: *p* = 0.001), suggesting a different role of the liver towards glutamate utilization relatively to glutamine. Taurine concentration is, similarly to glutamate, increased (or tended to) in two vessels in the fed state (PP time effect: *p* = 0.068 and *p* = 0.007 in artery and portal vein, respectively, not significant in the hepatic vein) (Figure 5). In the portal vein, a more important post prandial increased level of taurine is observed at D14 relatively to D1 (Day: *p* = 0.02) (Figure 5). No such profile existed for taurine both in artery and hepatic vein. Lastly, glycine presented a very specific profile (not seen for all other AA): an increased fasted level in the three vessels was observed both at D14 and D60 (*p* < 0.01 for all vessels) (Figure 5). Associated with this, in the fed state, glycine concentrations were decreased at D14 and D60 whereas glycine levels at D1 increased in all vessels (PP time effect: *p* < 0.001, *p* = 0.08 and *p* < 0.001 in artery, portal vein, and hepatic vein, respectively) (Figure 5).

## 4. Discussion

In the present study we assessed the effect of two-month overfeeding on nutrients uptake/release by the splanchnic area in the adult mini pig in the fasted state for a wide range of molecules and in the fed state for selected metabolites/molecules (glucose, lactate, urea, insulin, amino acids). We mention that the data presented in the fasted state at D1 represent the metabolic status of an animal adapted to a diet providing energy and proteins capable to maintain body weight stable and the post prandial state at D1 is the first day they receive the HFHS diet.

Although the animals did not present diabetes or fasting hyperglycemia at the end of the HFHS overfeeding period (and as already shown in a previous work on other animals but a rather similar diet [18]), their utilization of energy and nitrogenous nutrients at the splanchnic level was strongly altered to handle the unusual nutrients overflow both in the fed and the fasted states due to overfeeding. Some short term (after 14 days of overfeeding) and longer-term adaptive mechanisms are discussed in the present study.

### 4.1. Metabolic Adaptations to Overfeeding in Pigs: Impact on Nitrogenous and Energy Nutrients at the Gut, Hepatic, and Whole Body Levels

As the primary tissue in contact with the diet changes, the gut leaves large quantities of glucose to reach the portal vein when animals are fed the HFHS diet. As could be expected following the meal intake, the net plasma gut release is increased post-prandially as well as at the portal, hepatic and to a lesser extend arterial level from D1 to D60. These kinetic patterns are not differentially altered between D1 and D60, as shown by an absence of difference of significant net plasma gut release of glucose between D1 and D60. This is not the case in the fasted state where glucose concentrations are not altered in all vessels, but net plasma gut release increased, suggesting an intense utilization of glucose by other tissues and possibly by peripheral tissues, as generally observed in the fasted state (e.g., muscle and adipose tissues). The fact that the net glucose release by the liver and splanchnic area (Table 4) remains stable in the fasted state throughout time shows that contrarily to what generally occurs in well installed IR, no increased hepatic glucose release/production occurs in our model [26,27,28,29]. Residual amounts of glucose released by digestion of carbohydrates from the previous meal can be one of the explanations to the significant increased glucose net plasma gut release observed in the fasted state at D60 compared to D1. As HFHS meal supplies important amounts of nutrients, and particularly lipids, carbohydrates hydrolysis and digestion rate may be delayed between D1 (animals were adapted to the Control diet) and D14/D60 where the gut had been adapted to HFHS diet. Another possibility is an increased gluconeogenesis by the gut due to the increased supply of gluconeogenic precursors, as previously suggested [16], such as luminal amino acids (alanine for instance), plasma lactate or even small amounts of short chain fatty acids produced by microbiota from dietary fibers (tendency for an increased net gut release of propionate, suggesting an increased synthesis of SCFA by microbiota in the gut, Table 4). It should be noted that the increased overall food intake between D1 and D60 is associated with and increased supply of dietary fibers, potentially capable to stimulate microbiota activity. The increased SCFA synthesis by microbiota observed in the present study (as shown by net portal release of propionate) has already been reported in the literature on obesity (and related diseases) and illustrates the complex role of SFCA in the relationship between microbiota and host (SCFA increased both in fiber-supplemented [30] but also in obese individuals [31]). Indeed, as suggested by [31], according to their signaling pathway or metabolic mechanism they are involved in, they can either promote (via stimulation of triglycerides accumulation) or prevent (Histone deacetylases inhibition and/or GPR 41 and 43 activation) hepatic steatosis. In the present work, our hypothesis is that overfeeding (more than the relative oversupply of lipids) associated with an increased overall supply of dietary fibers may have maintained the activity of carbohydrates degrading microbes, leading to increased portal release of some SCFA.

To handle the important supply of nutrients, particularly glucose, and notably avoid even more massive glucose release, the gut adapts by oxidizing it into lactate, which is then released into the portal vein (more increased in the fed states (Figure 2) at D14 and D60 relative to D1). These increased lactate levels are visible in all vessels including artery. Such an increased fasting lactate level is known to occur progressively in obesity [32] and during IR installation [33], as observed in our model with D14 values intermediate between D1 and D60. As glucose supply from the diet is important due to overfeeding in the fed state, a conversion of glucose into pyruvate followed by its reduction into L-lactate (anaerobic glycolysis [34]) is a mechanism that can limit hyperglycemia [35]. Aside from a production in the gut, and as the portal vein also drains visceral adipose tissue, an increased lactate production from adipose tissues located at the gut level cannot be excluded [14]. In the fasted state, other mechanisms can explain the increased lactate levels. The sites of metabolic adaptation are also different (located at the muscle/adipose tissues and liver levels). The lactate that reaches the liver may not entirely be metabolized into glucose [35], and lactate net release from the liver has been shown to increase between D1 and D60, suggesting a limited capacity for lactate uptake (notably for gluconeogenesis).

The oversupply of food does not only disturb the energy metabolism but also amino acid utilization for energy, protein metabolism, and urea synthesis/elimination. First, levels of urea in all vessels are decreased both in the fasted state and the fed state. Such a decreased plasma urea content has already been discussed in a previous paper [19], in rodents fed a cafeteria diet [36] and corresponds to a nitrogen-sparing effect due to overfeeding [37]. The increased availability of all nutrients (lipids, glucose, AA, other N-based products, like nucleotides) tend to limit AA oxidation and, consequently, urea production. Due to this (and also the increased supply of proteins in HFHS-fed animals), a relatively small, but significant, increased AA plasma level (TAA, and among them particularly some NIAA (glycine) and BCAA) is observed. The reduced activity of the urea cycle observed is supported by the increased level of fasting plasma ornithine which accumulates instead of being metabolized into citrulline via carbamoyl-P synthetase and NH_3_ incorporation into the urea cycle. For technical reasons, we could not measure plasma ammonia levels but data from pigs fed a similar diet (unpublished results) did not present significant alterations of ammonia levels throughout the obesity development. This is also in line with the increased concentrations we observe for glycine and serine known to be direct precursors for ammonia synthesis [38], and which accumulate instead of being catabolized into ammonia (and later into urea). Hence, in absence of necessity to detoxify ammonia, urea cycle remains at a low level. Our present model of oversupply of both energy and AA ends up with a mechanism of decreased urea and probably ammonia synthesis and relative increased level of certain AA. This lies a question un-answered: where and under what form the nitrogen excess can be utilized? A stimulation of muscle growth and AA utilization for protein synthesis could occur in our pigs (even if adults) associated with an increase in animal size (as previously shown in a similar study [18]) and may help to avoid massive hyper-aminoacidemia. This could be mediated by insulin that is highly responsive to meal intake at D14, suggesting that growth stimulation might be maximum at D14 (very low urea/high AA levels at D14) and intermediate between D14 and D60 (insulin less stimulated by meal and urea levels intermediate Figure 2 and Figure 4). Other fates for nitrogen could be: N_2_ in expired air or urinary nitrates, nitrites, uric acid, peptides or amino acids. An increased N excretion as AA in urine is possible as the presence of AA in urine has been spotted in a recent work on the same model [18].

**Figure 6:**
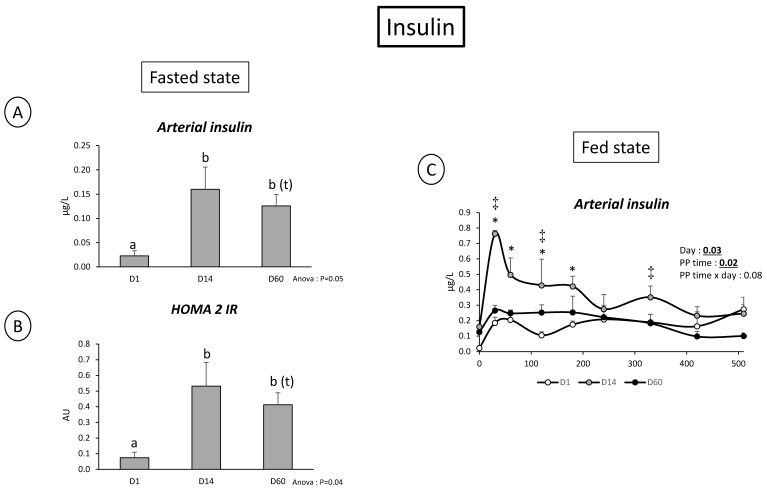
(**A**) Arterial insulin concentration (µg/L) in the fasted before (D1) and after 14 days (D14) and 60 days (D60) of adaptation to HFHS diet. (**B**) HOMA 2 IR (UA) in the fasted state before (D1) and after 14 days (D14) and 60 days (D60) of adaptation to HFHS diet. (**C**) Insulin concentration (µg/L) after meal intake (Post prandial state, PP: 0 to 510 min) in artery before (D1) and after 14 days (D14) and 60 days (D60) of adaptation to HFHS diet. Fasted state: One way RM ANOVA: with different letters: significantly different; (t): tendency. Fed state: Two way RM ANOVA, * D14 significantly different from D1, † D60 significantly different from D1, ‡ D60 significantly different from D14; Values are means ± SEM. For calculations and statistical treatment of the data and for details, see Materials and Methods.

Looking at specific amino acids, a significant alteration of post prandial profile of the glutamate/glutamine couple was observed in our study. Indeed, glutamine portal concentration was increased both at D14 and D60 vs. D1 (Figure 4). This suggests that this major fuel for small intestine (along with glutamate) [39] is replaced by other energy nutrients, a mechanism already demonstrated elsewhere [40]. In parallel, glutamate post prandial concentration in the portal vein was also significantly increased, but only at D14, whereas it was not altered at D1 and D60. One explanation is that glutamine, used by the gut as a fuel at D1, could have been replaced by glucose as a fuel at D14 and glutamate and glucose at D60. At D1, as it was the first time that the gut was challenged by the diet, catabolism of all energy nutrients was stimulated. Due to the long term overfeeding, at D14, a prioritization of glucose gut uptake and utilization may have occurred via a stimulation by insulin (increased in PP state at D14 (Figure 6)), as glucose uptake may be at least partially regulated by insulin receptors present in intestine epithelium, [41]. Lastly, the glutamate excess present in the enterocytes, due to glutamate dietary supply and potentially glutamine excess, could ultimately lead to an increased utilization of glutamate by the gut to limit overall hyperaminoacidemia. This increased utilization of glutamate by the gut and activation of Krebs cycle [42] could lead to an increased production of lactate, in absence of total catabolism, and concur to explain the increased lactate portal concentration and net plasma gut release observed at D60. This could also explain why urea levels are less decreased at D60 compared to D14 (D60 plasma concentrations were intermediate between D1 and D60).

Our dietary intervention was also characterized by an increased supply of lipids (palm oil) leading to an increased arterial cholesterol level (Table 1). Interestingly, glycine and taurine, two amino acids involved in recycling of biliary acids in human and pigs [43] presented an increased level in all vessels in the fasted state for glycine and in the portal vein in the fed state at D14 for taurine (Figure 5). As they combine to bile acids within the liver to be further recycled into the bile, their utilization in the liver is increased when dietary supply of lipids is increased. However, the fact that the increased availability is observed in the portal vein in the fasted state for glycine and in the fed state for taurine with a probable utilization within the liver for both AA, the metabolic fate of these two AA in relation to lipids intake should require further investigation. Interestingly, glycine has also been demonstrated, when administrated in the diet of rats, to limit non esterified fatty acids and lipids accumulation in adipocytes, via a stimulation of mitochondrial activity [44]. Aside from the already observed “buffering effect” of adipose tissues to limit hyperlipidemia [18]. Could the increased plasma glycine observed in the fasted state in our animals be another endogenous mechanism capable to counteract the increased plasma lipids concentration (in our study, triglycerides are not altered)?

### 4.2. Analysis of the Kinetics of Evolution of the Arterial Metabolites in Our Model of Overfed Mini Pigs

As already discussed in the present paper but also in previous published works [18], the metabolic shifts observed in our model represents the early stages of development of obesity and diet-induced metabolic disturbances ultimately leading to insulin resistance (IR). Increased arterial fasted cholesterol (total, LDL and HDL), lactate, insulin and HOMA2 IR are observed between D1 and D60. In parallel, short chain fatty acids (acetate, isobutyrate, or isovalerate) and several amino acids (AA): leucine, isoleucine, valine, tryptophane, glycine, ornithine, and proline increased, whereas urea and methionine decreased between D1 and D60.

However, the pattern of change of these parameters can differ. Some parameters are significantly modified only at D14 (arterial isobutyrate, isovalerate, and LDL cholesterol) and represent a short lasting diet-dependent adaptations. A second group of parameters are modified early (D14) and remained stable at D60 (AA: methionine, proline, isoleucine, glycine, phenylalanine, lactate, urea, fructosamine, insulin, HOMA, total and HDL cholesterol), suggesting that these alterations can be very closely linked to the rapid shift from a “maintenance” diet to a “high fat high sucrose” diet but could also be considered as very early markers of a metabolic shift and/or development of insulin resistance. Looking at the potential regulatory role of glycine on lipids and cholesterol handling (see above), this AA should be studied in detail. Lastly, some parameters (acetate, leucine, valine, tryptophane, and serine associated with the progressive pigs’ weight) are also particularly interesting as they are significantly (or tend to be) increased more progressively and more lately than the other parameters, suggesting a progressive shift of their utilization/metabolism at the whole body level (less driven by the direct impact of the change of diet and associated nutrients). Leucine and valine are already considered as predictors of insulin resistance [45] and their slow increase over the entire experimental period confirms their relevance in the development of insulin resistance/obesity phenotype [19]. Acetate is less studied but its concentration is increased in the caecum and plasma as well as its overall turnover in insulin resistant or high fat fed animals/humans [46,47,48]. The role of the microbiota in this increased acetate production of obese/IR states, particularly in the fasted state may be significant [46] and may lead to altered glucose-stimulated insulin production, fat storage and could ultimately lead to insulin resistance. Unfortunately, due to high variability between animals, no increased gut or splanchnic net acetate production could be observed in the present study (Table 4) whereas it is the case for propionate and butyrate. A reduction of acetate utilization by muscles for oxidation is a possible explanation for the increased arterial acetate levels, as a significant part of acetate utilization takes place in muscle [49]. The establishment of a resistance for acetate oxidation in muscle can be hypothesized as acetate oxidation has been shown insulin-sensitive in rodents’ hindquarters [50]. Lastly, tryptophan has also been shown (among aromatic amino acids), to be increased and tightly correlated to obesity, adiponectin and intrahepatic fat content [51,52,53].

In conclusion, we have shown that two months of overfeeding in pigs led to important metabolic shifts which may have led to the maintenance of a relatively stable glycemia. This was achieved thanks to important shifts in lactate and amino acids/urea metabolism. A progressive increased release of lactate by the gut and the liver in the fasted and the fed state observed in the present study could trigger other metabolic perturbations leading to nutritional-related pathologies and should be examined in detail. The analysis of the profile of arterial metabolites throughout the experimental period showed, as could be anticipated, a progressive increased BCAA levels (as detailed elsewhere [19]) but also acetate, tryptophan and, potentially, glycine, which are less studied in the context of obesity development, should require further investigation as potential markers of metabolic shift towards insulin resistance.

## Figures and Tables

**Table 1:** Animals’ weight, arterial concentration in the fasted state of glucose, lactate, urea, triglycerides, total cholesterol, Low Density Lipoprotein (LDL)-cholesterol, High Density Lipoprotein (HDL)-cholesterol, albumin, alanine aminotransferase (ALT), aspartate aminotransferase (AST) before (D1) and after 14 days (D14) and 60 days (D60) of adaptation to High Fat High Sugar (HFHS) diet. Average plasma flow in portal vein (PV), hepatic vein (HV), and hepatic artery (HA) from 0 to 8.5 h post meal ingestion before (D1) and after 14 days (D14) and 60 days (D60) of adaptation to HFHS diet.

Parameter	Vessel	D1	D14	D60	ANOVA
Pig’s weight		31.52 ± 1.49 a	35.70 ± 1.51 b	45.16 ± 1.56 c	<0.001
Kg					
Glucose	A	5.20 ± 0.35	5.46 ± 0.29	5.26 ± 0.11	0.85
mmol/L					
Lactate	A	0.55 ± 0.05 a	0.86 ± 0.94 b	0.85 ± 0.05 b(t)	0.03
mmol/L					
Urea	A	5.34 ± 0.43 a	3.83 ± 0.31 b	4.04 ± 0.31 b(t)	0.03
mmol/L					
Triglycerides	A	0.19 ± 0.01	0.25 ± 0.04	0.24 ± 0.05	0.15
mmol/L					
Total	A	1.76 ± 0.10 a	2.79 ± 0.09 b	2.53 ± 0.16 b	<0.001
Cholesterol					
mmol/L					
LDL	A	0.97 ± 0.03 a	1.30 ± 0.02 b	1.00 ± 0.08 a	<0.001
Cholesterol					
mmol/L					
HDL	A	0.67 ± 0.03 a	1.28 ± 0.09 b	1.06 ± 0.08 c	<0.001
Cholesterol					
mmol/L					
Albumin	A	536 ± 20	544 ± 14	537 ± 17	0.70
µmol/L					
ALT	A	29.7 ± 0.8	27.1 ± 1.8	25.4 ± 1.7	0.15
U/L					
AST	A	36.2 ± 4.2	37.9 ± 5.2	28.8 ± 2.3	0.36
U/L					
Plasma flow	PV	0.60 ± 0.05	0.69 ± 0.05	0.66 ± 0.03	0.37
L/min					
Plasma flow	HV	0.78 ± 0.03	0.82 ± 0.08	0.84 ± 0.01	0.80
L/min					
Plasma flow	HA	0.17 ± 0.02	0.13 ± 0.03	0.18 ± 0.03	0.34
L/min					

Part of the data (D1 and D60) for glucose, lactate, triglycerides, cholesterol, and urea are published elsewhere [18]. Values are means ± SEM; for details on the statistical treatment of the data see Materials and Methods.

**Table 2:** Acetate (C2), propionate (C3), butyrate (C4), isobutyrate, isovalerate and C2+C3+C4 concentrations in the fasted state in artery (A), portal vein (PV), and hepatic vein (HV) before (D1) and after 14 days (D14) and 60 days (D60) of adaptation to HFHS diet.

Metabolite	Vessel	D1	D14	D60	ANOVA
Acetate (C2)	A	552.3 ± 14.5 a	572.1 ± 34.6 a	761.8 ± 50.0 b	0.02
µmol/L	PV	844.8 ± 53.8	897.0 ± 54.7	1000.2 ± 25.5	0.07
	HV	744.5 ± 53.1	861.3 ± 81.2	896.2 ± 41.0	0.33
Propionate (C3)	A	22.7 ± 0.5	26.8 ± 4.4	27.1 ± 4.4	0.50
µmol/L	PV	199.8 ± 22.6	232.0 ± 30.2	229.5 ± 16.7	0.50
	HV	26.1 ± 1.2 a	28.0 ± 2.7 ab	32.0 ± 4.4 b(t)	0.05
Butyrate (C4)	A	2.0 ± 0.6	5.0 ± 1.4	2.8 ± 1.1	0.30
µmol/L	PV	63.0 ± 6.7	80.7 ± 11.1	81.0 ± 4.3	0.09
	HV	14.4 ± 1.7	19.9 ± 6.5	15.4 ± 2.1	0.62
Isobutyrate	A	2.3 ± 0.1 ab	1.9 ± 0.19 b	2.5 ± 0.2 a	0.03
µmol/L	PV	15.3 ± 2.1 a	11.1 ± 1.2 b(t)	12.8 ± 0.2 ab	0.08
	HV	3.0 ± 0.2	1.8 ± 0.6	2.8 ± 0.3	0.20
Isovalerate	A	1.8 ± 0.1 a	1.6 ± 0.1 b	2.0 ± 0.1 c	0.002
µmol/L	PV	8.7 ± 1.4	7.0 ± 0.6	8.3 ± 0.2	0.12
	HV	2.3 ± 0.3	2.5 ± 0.5	2.6 ± 0.2	0.37
C2+C3+C4	A	577 ± 17 a	603 ± 40 a	792 ± 55 b	0.02
µmol/L	PV	1108 ± 81	1210 ± 91	1311 ± 32	0.09
	HV	851 ± 50	909 ± 88	944 ± 37	0.46

Values are means ± SEM; for details on the statistical treatment of the data see Materials and Methods.

**Table 3:** Amino acids concentrations (µmol/L) in the fasted state in artery (A), portal vein (PV) and hepatic vein (HV) before (D1) and after 14 days (D14) and 60 days (D60) of adaptation to HFHS diet.

Metabolite	Vessel	D1	D14	D60	ANOVA
Leucine	A	145.2 ± 8.3 a	146.9 ± 5.8 a	172.0 ± 9.6 b	0.03
µmol/L	PV	149.0 ± 10.5 a	151.1 ± 7.3 a	204.0 ± 7.2 b	0.01
	HV	150.2 ± 8.9 a	161.0 ± 9.8 a	190.8 ± 12.5 b(t)	0.06
Isoleucine	A	102.9 ± 8.0 a	126.3 ± 2.6 b	120.8 ± 2.2 b(t)	0.03
µmol/L	PV	103.8 ± 10.7 a	127.4 ± 2.5 ab	139.3 ± 5.6 b(t)	0.06
	HV	104.2 ± 9.5 a	137.8 ± 7.5 b(t)	134.0 ± 7.0 b(t)	0.05
Valine	A	275.2 ± 14.5 a	276.7 ± 12.0 a	302.8 ± 16.9 b(t)	0.06
µmol/L	PV	276.0 ± 15.0 a	275.8 ± 11.8 a	336.5 ± 5.2 b	0.02
	HV	279.8 ± 14.4 a	285.3 ± 10.0 a	323.4 ± 16.5 b(t)	0.06
Lysine	A	139.5 ± 14.4	157.7 ± 18.5	166.4 ± 5.0	0.37
µmol/L	PV	143.4 ± 18.5	164.2 ± 12.3	193.5 ± 10.0	0.24
	HV	134.0 ± 15.7	167.8 ± 9.8	171.8 ± 9.9	0.08
Phenylalanine	A	56.8 ± 2.1 a	47.2 ± 2.0 b(t)	53.0 ± 2.0 ab	0.06
µmol/L	PV	58.8 ± 3.3	52.4 ± 3.7	66.0 ± 2.8	0.12
	HV	54.4 ± 2.8	51.0 ± 3.0	53.2 ± 1.8	0.68
Methionine	A	26.4 ± 2.3 a	19.0 ± 0.8 b	19.0 ± 0.4 b	0.02
µmol/L	PV	26.0 ± 3.1	19.8 ± 0.9	22.8 ± 1.0	0.25
	HV	22.2 ± 2.2	21.0 ± 1.5	19.8 ± 0.9	0.65
Threonine	A	132.8 ± 13.2	123.2 ± 7.8	135.3 ± 4.9	0.43
µmol/L	PV	129.8 ± 12.2	123.6 ± 5.3	151.3 ± 6.9	0.25
	HV	129.4 ± 9.4	123.0 ± 5.4	130.8 ± 6.6	0.97
Tryptophane	A	28.0 ± 2.0 a	30.8 ± 1.4 a	35.8 ± 1.9 b(t)	0.09
µmol/L	PV	27.8 ± 2.2 a	27.6 ± 1.4 a	36.0 ± 2.8 b(t)	0.07
	HV	27.0 ± 2.0 a	35.5 ± 1.0 b(t)	32.2 ± 2.7 ab	0.05
Histidine	A	74.0 ± 4.2	69.4 ± 2.5	76.3 ± 1.4	0.34
µmol/L	PV	75.0 ± 5.9	72.0 ± 2.2	84.8 ± 1.4	0.28
	HV	71.0 ± 5.2	70.5 ± 1.0	78.0 ± 2.0	0.39
Alanine	A	202.8 ± 31.5	216.4 ± 24.4	222.3 ± 19.6	0.93
µmol/L	PV	233.0 ± 42.7	268.8 ± 26.4	286.0 ± 19.1	0.68
	HV	158.0 ± 32.2	224.3 ± 21.5	215.4 ± 12.2	0.20
Glutamate	A	129.6 ± 15.4	156.8 ± 12.3	140.8 ± 15.7	0.48
µmol/L	PV	120.2 ± 16.9	130.4 ± 9.5	131.0 ± 19.3	0.90
	HV	280.8 ± 44.0	279.8 ± 53.2	357.4 ± 42.2	0.41
Glutamine	A	244.5 ± 13.8	215.3 ± 24.2	213.5 ± 16.2	0.46
µmol/L	PV	200.4 ± 12.6	179.4 ± 12.4	183.3 ± 15.6	0.50
	HV	196.4 ± 11.7	190.3 ± 9.9	181.3 ± 15.3	0.75
Glycine	A	624.4 ± 72.2 a	864.6 ± 51.9 b	785.3 ± 39.2 b	0.01
µmol/L	PV	650.2 ± 84.0 a	977.2 ± 59.5	953.3 ± 19.5	0.007
	HV	620.0 ± 78.2 a	894.3 ± 57.3 b	824.0 ± 41.5	0.001
Serine	A	133.8 ± 7.2 a	149.8 ± 4.3 ab	165.8 ± 15.4 b(t)	0.09
µmol/L	PV	130.4 ± 10.8 a	152.6 ± 5.7 b(t)	182.8 ± 13.0 c	0.009
	HV	132.4 ± 11.1	156.5 ± 10.6	158.4 ± 11.5	0.11
Tyrosine	A	66.4 ± 7.7	62.0 ± 7.5	76.5 ± 12.2	0.28
µmol/L	PV	66.4 ± 7.0 a	66.2 ± 9.3 ab	79.0 ± 14.2 b(t)	0.09
	HV	60.0 ± 7.2	69.3 ± 12.0	68.2 ± 9.0	0.17
Cystine	A	24.4 ± 2.6	22.2 ± 2.1	26.0 ± 0.9	0.66
µmol/L	PV	26.0 ± 3.8	25.2 ± 1.2	29.5 ± 0.5	0.74
	HV	27.0 ± 3.3	23.0 ± 0.9	24.4 ± 2.3	0.75
Citrulline	A	69.6 ± 5.9	77.4 ± 4.9	70.0 ± 4.3	0.38
µmol/L	PV	86.2 ± 8.3	92.8 ± 4.7	97.5 ± 5.3	0.52
	HV	78.6 ± 6.6	92.3 ± 6.4	89.8 ± 5.1	0.13
Ornithine	A	54.6 ± 4.7 a	76.2 ± 7.6 b(t)	68.0 ± 4.3 ab	0.06
µmol/L	PV	61.8 ± 6.3	83.2 ± 8.9	77.8 ± 5.1	0.17
	HV	59.6 ± 6.1 a	86.5 ± 6.1 b	75.6 ± 4.2 ab	0.04
Arginine	A	87.8 ± 6.0	87.4 ± 9.2	100.8 ± 4.6	0.60
µmol/L	PV	86.0 ± 12.0	93.4 ± 8.7	114.5 ± 4.5	0.32
	HV	80.6 ± 9.4	98.8 ± 7.0	102.0 ± 4.2	0.20
Taurine	A	72.7 ± 2.3	75.1 ± 4.5	73.8 ± 2.4	0.85
µmol/L	PV	76.3 ± 2.7	76.0 ± 4.3	74.0 ± 4.8	0.81
	HV	77.1 ± 3.0	80.7 ± 5.6	74.8 ± 3.8	0.78
Proline	A	215.8 ± 33.3 a	379.2 ± 10.7 b	379.0 ± 9.8 b	0.003
µmol/L	PV	208.4 ± 26.5 a	381.4 ± 6.6 b	379.3 ± 40.6 b	0.006
	HV	229.8 ± 35.7 a	394.8 ± 15.5 b	402.6 ± 7.7 b	0.002
3-methyl histidine	A	24.2 ± 0.6 ab	21.2 ± 2.5 a	28.5 ± 2.2 b(t)	0.09
µmol/L	PV	24.2 ± 0.8	24.2 ± 1.2	25.3 ± 5.0	0.99
	HV	26.0 ± 2.2	22.0 ± 2.4	29.6 ± 2.1	0.13
Carnosine	A	16.8 ± 1.9	20.2 ± 2.3	18.5 ± 2.2	0.17
µmol/L	PV	17.8 ± 2.2	19.4 ± 1.5	18.0 ± 1.2	0.72
	HV	20.6 ± 2.1	22.0 ± 2.9	20.4 ± 2.2	0.98
BCAA	A	523.3 ± 29.2 a	549.8 ± 19.4 ab	595.6 ± 28.5 b(t)	0.07
µmol/L	PV	528.8 ± 35.3 a	554.3 ± 20.0 a	679.8 ± 16.2 b	0.03
	HV	534.2 ± 31.6 a	581.0 ± 24.3 ab	648.2 ± 35.0 b(t)	0.06
IAA	A	985.9 ± 57.8	997.1 ± 41.8	1081.2 ± 32.9	0.22
µmol/L	PV	989.6 ± 73.7	1009.9 ± 43.85	1234.0 ± 33.4	0.09
	HV	972 ± 58.8	1044.5 ± 38.6	1107.8 ± 48.9	0.19
NIAA	A	1877.9 ± 102.3 a	2330.3 ± 120.7 b	2276.2 ± 14.3 b(t)	0.03
µmol/L	PV	1873.2 ± 145.9 a	2474.8 ± 123.8 b	2539.1 ± 85.7 b	0.01
	HV	1929.2 ± 165.4 a	2530.5 ± 132.7 b	2528.7 ± 95.3 b	0.01
TAA	A	2742.4 ± 147.0 a	3198.4 ± 157.4 a	3210.9 ± 44.8 b(t)	0.07
µmol/L	PV	2771.0 ± 205.9 a	3352.3 ± 161.7 b(t)	3609.0 ± 88.2 b (t)	0.04
	HV	2789.8 ± 207.2 a	3440.7 ± 160.5 b	3498.7 ± 139.7 b	0.03

Branched chain amino acids (BCAA): leucine + isoleucine + valine; Indispensable amino acids (IAA): leucine + isoleucine + valine + lysine + phenylalanine + methionine + threonine + histidine + tryptophane; Non indispensable amino acids (NIAA): alanine + glutamate + glutamine + glycine + tyrosine + citrulline + cystine + 3 methyl-histidine + ornithine + arginine + proline + serine; Total amino acids (TAA): NIAA + IAA. Values are means ± SEM; for details on the statistical treatment of the data see Materials and Methods.

**Table 4:** Net gut, liver and splanchnic (gut + liver) fluxes of acetate (C2), propionate (C3), butyrate (C4), isobutyrate, isovalerate and C2+C3+C4 in the fasted state before (D1), 14 days (D14) and 60 days (D60) of adaptation to the HFHS diet.

Metabolite		D1	D14	D60	ANOVA
Glucose	Gut	6.84 ± 6.67 a	22.45 ± 6.31 a	33.41 ± 5.78 b(t)	0.05
mmol/h	Liver	29.78 ± 7.32	31.60 ± 2.65	36.31 ± 11.17	0.51
	Splanchnic	34.72 ± 12.18	50.81 ± 10.23	59.54 ± 15.16	0.56
Lactate	Gut	4.85 ± 1.49	1.45 ± 1.14	2.04 ± 0.73	0.17
mmol/h	Liver	−1.78 ± 3.08 a	2.90 ± 4.15 a	8.53 ± 0.63 b	0.03
	Splanchnic	2.85 ± 2.36	3.63 ± 4.36	10.57 ± 0.77	0.09
Urea	Gut	1.78 ± 0.89	5.74 ± 1.92	2.24 ± 3.49	0.47
mmol/h	Liver	7.11 ± 2.45	3.36 ± 1.47	6.60 ± 2.57	0.49
	Splanchnic	7.10 ± 1.55	9.69 ± 0.94	9.31 ± 2.29	0.32
Acetate	Gut	10,645 ± 1736	12,921 ± 1738	8717 ± 1563	0.47
µmol/h	Liver	−722 ± 2416	689 ± 1607	−418 ± 1205	0.42
	Splanchnic	9984 ± 1916	14,635 ± 3153	8299 ± 2739	0.26
Propionate	Gut	6193 ± 688	8210 ± 1038	8227 ± 664	0.09
µmol/h	Liver	−6447 ± 817	−8822 ± 622	−8191 ± 1205	0.11
	Splanchnic	164 ± 55	252 ± 119	35 ± 130	0.57
Butyrate	Gut	2176 ± 296 a	3048 ± 295 b(t)	3121 ± 189 b(t)	0.05
µmol/h	Liver	−1583 ± 357	−2362 ± 280	−2515 ± 187	0.23
	Splanchnic	581 ± 84	747 ± 216	607 ± 122	0.79
Isobutyrate	Gut	465 ± 86	375 ± 46	414 ± 29	0.37
µmol/h	Liver	−428 ± 99	−386 ± 32	−409 ± 27	0.64
	Splanchnic	31 ± 16	4 ± 29	5 ± 16	0.71
Isovalerate	Gut	252 ± 51	218 ± 18	253 ± 13	0.56
µmol/h	Liver	−227 ± 53	−177 ± 15	−229 ± 20	0.54
	Splanchnic	27 ± 15	44 ± 17	24 ± 9	0.85
C2+C3+C4	Gut	19,014 ± 2608	24,179 ± 2961	20,065 ± 816	0.50
µmol/h	Liver	−8476 ± 4498	−10,494 ± 961	−11,124 ± 1948	0.87
	Splanchnic	12,173 ± 1550	15,634 ± 3346	8942 ± 2720	0.40

Values are means ± SEM; for details on the calculations and statistical treatment of the data see Materials and Methods.

**Table 5:** Net gut, liver and splanchnic (gut + liver) fluxes of amino acids in the fasted state before (D1) and after 14 days (D14) and 60 days (D60) of adaptation to HFHS diet.

Metabolite	Area	D1	D14	D60	ANOVA
Leucine	Gut	147 ± 227	188 ± 133	793 ± 37	0.28
µmol/h	Liver	152 ± 84	343 ± 566	−71 ± 167	0.93
	Splanchnic	246 ± 182	620 ± 531	586 ± 194	0.86
Isoleucine	Gut	37 ± 143	46 ± 83	423 ± 21	0.27
µmol/h	Liver	71 ± 61	290 ± 367	−13 ± 141	0.84
	Splanchnic	70 ± 116	388 ± 357	343 ± 120	0.76
Valine	Gut	46 ± 265	−38 ± 71	619 ± 159	0.20
µmol/h	Liver	291 ± 75	446 ± 625	54 ± 237	0.97
	Splanchnic	238 ± 254	427 ± 592	623 ± 156	0.90
Lysine	Gut	154 ± 218	248 ± 110	816 ± 47	0.22
µmol/h	Liver	−341 ± 90	−31 ± 407	−948 ± 111	0.24
	Splanchnic	−238 ± 186	280 ± 391	−160 ± 101	0.55
Phenylalanine	Gut	81 ± 142	230 ± 131	563 ± 104	0.37
µmol/h	Liver	−161 ± 89	−145 ± 207	−512 ± 27	0.36
	Splanchnic	−101 ± 110	−157 ± 125	−1 ± 75	0.51
Methionine	Gut	−9 ± 61	2 ± 24	121 ± 26	0.61
µmol/h	Liver	−157 ± 92	84 ± 71	−105 ± 62	0.53
	Splanchnic	−176 ± 108	110 ± 44	−1 ± 35	0.1
Threonine	Gut	−64 ± 198	−8 ± 119	359 ± 37	0.55
µmol/h	Liver	20 ± 268	−6 ± 274	−639 ± 240	0.3
	Splanchnic	−97 ± 423	43 ± 328	−307 ± 106	0.76
Tryptophane	Gut	−3 ± 32 ab	−123 ± 30 b	111 ± 68 a	0.03
µmol/h	Liver	−29 ± 10 ab	305 ± 136 b	−328 ± 218 a(t)	0.05
	Splanchnic	−43 ± 31	166 ± 139	−189 ± 163	0.1
Histidine	Gut	42 ± 114	105 ± 42	319 ± 37	0.34
µmol/h	Liver	−145 ± 30	−35 ± 129	−288 ± 90	0.38
	Splanchnic	−132 ± 110	97 ± 110 103	36 ± 110 58	0.47
Alanine	Gut	1128 ± 583	2141 ± 447	2917 ± 719	0.23
µmol/h	Liver	−3106 ± 680	−2776 ± 1204	−2989 ± 1263	0.93
	Splanchnic	−2065 ± 837	−318 ± 965	−810.4 ± 863	0.33
Glutamate	Gut	−357 ± 268 a	−1023 ± 228 b(t)	−567 ± 72 a	0.04
µmol/h	Liver	6387 ± 1315	7692 ± 2556	9063 ± 273	0.68
	Splanchnic	5976 ± 1502	6531 ± 2759	8926 ± 434	0.61
Glutamine	Gut	−1545 ± 265	−1612 ± 547	−1628 ± 248	0.90
µmol/h	Liver	−468 ± 673	157 ± 413	−440 ± 395	0.53
	Splanchnic	−2106 ± 757	−1460 ± 1079	−1888 ± 208	0.89
Glycine	Gut	1049 ± 884 a	4605 ± 978 b(t)	5072 ± 1916 ab	0.08
µmol/h	Liver	−1003 ± 373	−4675 ± 1412	−4549 ± 2028	0.31
	Splanchnic	−170 ± 669	744 ± 977	562 ± 232	0.74
Serine	Gut	−110 ± 211	70 ± 246	428 ± 64	0.52
µmol/h	Liver	107 ± 84	−99 ± 421	−810 ± 193	0.32
	Splanchnic	−52 ± 288	103 ± 509	−430 ± 104	0.58
Tyrosine	Gut	7 ± 150	151 ± 89	371 ± 62	0.29
µmol/h	Liver	−259 ± 96	−167 ± 238	−605 ± 160	0.51
	Splanchnic	−279 ± 141	21 ± 250	−251 ± 79	0.64
Cystine	Gut	49 ± 123	120 ± 35	168 ± 47	0.79
µmol/h	Liver	57 ± 137	−141 ± 101	−350 ± 135	0.39
	Splanchnic	98 ± 208	−46 ± 114	−124 ± 93	0.94
Citrulline	Gut	580 ± 107	662 ± 146	1029 ± 44	0.19
µmol/h	Liver	−161 ± 202	−88 ± 221	−167 ± 130	0.82
	Splanchnic	396 ± 184	581 ± 405	920 ± 108	0.38
Ornithine	Gut	230 ± 84	293 ± 77	366 ± 48	0.79
µmol/h	Liver	5 ± 61	−99 ± 115	−68 ± 62	0.54
	Splanchnic	261 ± 112	225 ± 61	315 ± 38	0.36
Arginine	Gut	−75 ± 231	234 ± 41	638 ± 74	0.14
µmol/h	Liver	−218 ± 120	−3 ± 181	−623 ± 96	0.18
	Splanchnic	−324 ± 192	226 ± 184	38 ± 119	0.34
Taurine	Gut	149 ± 80	24 ± 44	132 ± 162	0.65
µmol/h	Liver	144 ± 67	185 ± 170	22 ± 181	0.75
	Splanchnic	223 ± 75	117 ± 121	225 ± 105	0.25
Proline	Gut	−237 ± 360	−14 ± 370	108 ± 2154	0.97
µmol/h	Liver	955 ± 1483	555 ± 1110	695 ± 2464	0.21
	Splanchnic	653 ± 1598	472 ± 1434	811 ± 376	0.95
3-methyl	Gut	1 ± 20	47 ± 31	28 ± 14	0.27
histidine	Liver	75 ± 97	−102 ± 108	22 ± 18	0.21
µmol/h	Splanchnic	67 ± 94	−73 ± 73	38 ± 24	0.31
Carnosine	Gut	39 ± 17	−17 ± 67	40 ± 79	0.63
µmol/h	Liver	162 ± 144	101 ± 96	25 ± 73	0.77
	Splanchnic	194 ± 147	44 ± 49	144 ± 57	0.47
BCAA	Gut	230 ± 629	196 ± 279	1834 ± 147	0.23
µmol/h	Liver	553 ± 203	1079 ± 1556	−29 ± 544	0.93
	Splanchnic	533 ± 543	1435 ± 1474	1552 ± 448	0.88
IAA	Gut	432 ± 1166	549 ± 586	4123 ± 299	0.17
µmol/h	Liver	−300 ± 618	1120 ± 2610	−5185 ± 1781	0.28
	Splanchnic	−235 ± 1239	2026 ± 2115	−816 ± 1210	0.61
NIAA	Gut	720 ± 2330	5674 ± 1476	8962 ± 5024	0.14
µmol/h	Liver	2371 ± 2231	257 ± 4121	−821 ± 6378	0.93
	Splanchnic	2409 ± 3985	7005 ± 3836	8118 ± 1275	0.64
TAA	Gut	1307 ± 3243	6141 ± 1766	12,704 ± 5186	0.15
µmol/h	Liver	2099 ± 1873	1542 ± 6094	−5203 ± 6534	0.80
	Splanchnic	2400 ± 4524	8945 ± 5525	7822 ± 1020	0.77

Branched chain amino acids (BCAA): leucine + isoleucine + valine; Indispensable amino acids (IAA): leucine + isoleucine + valine + lysine + phenylalanine + methionine + threonine + histidine + tryptophane; Non indispensable amino acids (NIAA): alanine + glutamate + glutamine + glycine + tyrosine + citrulline + cystine + 3 methyl-histidine + ornithine + arginine + proline + serine; Total amino acids (TAA): NIAA + IAA. Values are means ± SEM; for details on the calculations and statistical treatment of the data see Materials and Methods.
